# An Analysis of the Clinical Efficacy of Early Dynamic Orthosis after Finger Extensor Digitorum Rupture

**DOI:** 10.1155/2022/1267747

**Published:** 2022-06-26

**Authors:** Guiwei Lu, Xu Sun, Jijianxiong Cao, Shifeng Han, Su Jiang

**Affiliations:** ^1^Department of Rehabilitation, Taizhou People's Hospital, Taizhou, Jiangsu 225300, China; ^2^Department of Hand and Foot Surgical, Taizhou People's Hospital, Taizhou, Jiangsu 225300, China

## Abstract

**Objective:**

The main objective is to compare the clinical efficacy of the early use of dynamic orthosis in patients with a finger extensor digitorum rupture.

**Methods:**

A total of 50 patients with hand and foot trauma who received surgical treatment in our hospital from March 2017 to February 2021 were selected, and two patients were excluded from the study. The patients were randomly divided into two groups. The control group (group A) was treated with plaster fixation and routine rehabilitation, and the study group (group B) underwent dynamic low-temperature thermoplastic plate fixation and routine rehabilitation. Total active motion (TAM) and total passive motion (TPM) of the injured finger before treatment, one month after treatment, two months after treatment, and after corrective treatment were compared.

**Results:**

After treatment following a finger extensor digitorum rupture, the TAM and TPM of the injured fingers increased significantly in both groups. The TAM and TPM in group B were significantly better than those in group A after one and two months of treatment (*P* < 0.05). After two months of treatment, the rates of improvement in TAM and TPM in group B were significantly higher than those in group A.

**Conclusion:**

The early use of dynamic orthosis can significantly improve the motor function and motion amplitude of the injured finger, increase the elasticity and extension of the finger extensor digitorum, and promote the recovery of muscle strength. It is an effective corrective method for tendon contracture after finger extensor digitorum rupture and has great value in clinical application.

## 1. Introduction

A finger extensor digitorum injury is a common clinical disease with many pathogenic factors, with the most common being violent injury or cutting injury. After a tendon injury, the development of a fibrous healing response impairs tendon function and limits the movement of the tendon [1]. At present, surgery and conservative treatment are the most common treatment methods, and tendon tissue fibrosis is the main clinical problem following hand surgery [1]. Clinical studies have shown that many patients may develop finger joint contracture, tendon adhesion, joint activity limitation, and even hand deformity after treatment, which seriously affects hand function and significantly reduces the patient's ability to perform activities of daily living and quality of life [2]. The roughness, adhesion, and stiffness of the tendon surface and the fibrosis of the tendon sheath affect tendon sliding and joint motion [1, 3, 4]. Therefore, maintaining the integrity of the tendon sheath and inhibiting exogenous tendon healing play an important role in preventing tendon adhesion and improving tendon activity [5]. In the past, surgeons and therapists used short splints or plaster to immobilize the finger joint, which can induce joint stiffness, contracture, and muscle atrophy. However, early tendon sliding can greatly reduce the incidence of tendon adhesion, contracture, and secondary release [6]. To ensure improved active finger motion function, the ideal tendon repair should provide extensive support for postoperative rehabilitation and minimize the occurrence of complications. Therefore, early intervention is important [2, 7].

The low-temperature thermoplastic plate has good plasticity and can be used to create and install a new orthopedic instrument for external fixation using dynamic orthosis. Dynamic orthosis adopts the principle of stress relaxation. In order to increase the range of joint motion, self-regulation of the angle of tension is achieved by adjusting the tension of the steel wire spring according to the patient's condition and situation in order to reduce secondary damage caused by excessive drafting of gypsum [8]. Using a randomized controlled study, this experiment will analyze and discuss the clinical efficacy of the early use of dynamic orthosis in patients with a finger extensor digitorum rupture in order to improve the rehabilitation treatment method for finger extensor digitorum rupture and provide a better theoretical basis for the formulation of a clinical rehabilitation treatment plan.

## 2. Materials and Methods

### 2.1. General Data

In this study, 50 patients with finger extensor digitorum rupture who were hospitalized for surgical treatment in the Department of Hand and Foot Surgery of our hospital from March 2017 to February 2021 were selected. Two of them were excluded from the group due to rerupture of the extensor tendon due to self-extension during early treatment. They were randomly divided into group A (*n* = 24) and group B (*n* = 24) according to the random number table method. There was no statistically significant difference in gender and age between the two groups (*P* > 0.05), indicating comparability (see Table 1). The experiment was approved by the Ethics Committee of the Jiangsu Taizhou People's Hospital, and all patients signed informed consent.

### 2.2. Inclusion and Exclusion Criteria

Inclusion criteria were as follows: (1) finger extensor digitorum injury, (2) admitted for surgery within a week of the rupture, (3) no fracture in the hand, (4) according to the Verdan method, we selected the patients in zone II to VI finger extensor digitorum injury, and (4) conscious and cooperative.

Exclusion criteria were as follows: (1) zone I finger extensor digitorum injury, (2) finger extensor digitorum rupture combined with flexor tendon rupture, (3) presented at the hospital more than one week after the rupture, or (4) finger extensor digitorum rupture with median ulnar and radial nerve injury. Patients were randomly divided into group A (18 males and 6 females) and group B (18 males and 6 females). There was no significant difference in basic information between the two groups (*P* > 0.05), indicating that the two groups were comparable.

## 3. Methods

### 3.1. Therapeutic Method

Group A was treated with plaster fixation and routine rehabilitation. These patients were treated with static fixation using conventional plaster after surgery, and the dressing was changed regularly to observe the healing of the affected hand. Four weeks later, the external fixation was removed, and the patient was instructed to move the fingers actively and passively and perform finger tendon sliding, tendon pulling, and hand function exercises.

Group B underwent dynamic orthosis and routine rehabilitation. An external dynamic orthotic was prepared and installed after the first postoperative dressing change. During the period of dynamic orthosis, the patient was instructed to actively flex the finger within the finger limit, and the finger was passively pulled and stretched by the wire. During the fixation period, 3–5 groups of active finger flexion were performed (20 times in each group) every 2 h during the day. According to the patient's tolerance, they were not allowed to actively extend the finger or passively straighten the finger at night to avoid active movement. The tension of the steel wire spring was regularly adjusted according to the patient's recovery progress. Within four weeks, the flexion angle of the metacarpophalangeal joint was controlled at about 40°. After four weeks, the fixation was removed, and active and passive finger movements were performed to guide the patient's finger tendon sliding and pulling and hand function exercises.

The single-blind method was used in this study, the same occupational therapist was responsible for the treatment of the patients in both groups, and another occupational therapist was responsible for the evaluation before, during, and after treatment.

### 3.2. Observation Index

The range of motion of the finger was measured in all enrolled patients using a joint angle ruler (metacarpophalangeal joint, proximal interphalangeal joint, and distal interphalangeal joint). The measurements were taken before surgery, one month after surgery, and two months after surgery and included the total active motion (TAM) and total passive motion (TPM) of the injured finger, in accordance with the standard methods developed by the Tendon Injury Committee of the International Federation of Hand Surgery (see Table 2). The larger the angle of TAM and TPM, the higher the rate of excellent and good, and the better the effect of therapy.

## 4. Statistical Analysis

SPSS 26.0 was used for statistical analysis. The enumeration data were expressed as frequency or percentage, and the chi-squared or rank-sum test was used. The measurement data were expressed as mean ± standard deviation ($\bar{x}\pm s$), and the independent sample *T*-test or one-way analysis of variance was used. The improvement rates were compared using the rank-sum test and chi-squared test. *P* < 0.05 was considered statistically significant.

## 5. Results

There was no significant difference in the TAM and TPM between the two groups before treatment (*P* > 0.05); therefore, finger joint mobility could be compared before treatment. During treatment, the TAM and TPM increased gradually in both groups (*P* < 0.05). When compared with group A, the change in TAM and TPM after one and two months was significantly higher in group B (*P* < 0.05; see Table 3). After two months of treatment, the improvement rates of TAM and TPM in group B were higher than those in group A (*P* < 0.05; see Table 4).

## 6. Discussion

With continuous developments in modern life, the function of the hand is increasingly important. Tendons are responsible for controlling finger movement, and the degree of sliding of tendons affects the movement ability of the fingers. After finger extensor digitorum injury, the anatomical continuity of the tendon sheathing canal is lost, and both external and internal healings are required in the process of postoperative finger extensor digitorum healing. External healing can easily affect tendon healing, resulting in tendon adhesion, finger joint stiffness, and limited finger flexion and extension activities. In order to avoid subsequent tendon rupture, the conventional treatment is to fix the finger with static plaster for four weeks following surgery. When considering the principle of muscle movement, this conventional treatment causes muscles and joints to be static for a long time, and the patient is unable to conduct early functional exercises, which can cause tendon adhesion, joint contracture, and a serious loss of hand function. Early active finger injury is easy to cause tendon fracture again, so it is necessary to fully communicate with the surgeon and the patient when installing the dynamic brace and strictly implement “active flexion and passive extension”. Secondly, the early dynamic brace we made and used has a barrier, so the range of finger movement can be limited according to needs. The wire spring can control the direction of finger movement and the passive tensile strength, which can effectively limit the phenomenon of tendon elongation. In order to improve the hand function of patients, the objective of this study is to compare the influence of static plaster fixation and dynamic low-temperature thermoplastic plate fixation on the prognosis of patients after finger extensor digitorum rupture. There are many factors affecting finger function after surgery, for example, finger tissue edema, joint–tendon adhesion, joint capsule contracture, and muscle weakness. Successful surgery is the basis for recovery, and timely, reasonable, and effective functional exercise is the key to the recovery of hand function [9]. Early postoperative finger functional exercise is a combination of active and passive training starting as soon as possible after surgery. It can accelerate tissue circulation, improve tissue edema, increase tendon tension, and promote tendon healing [10]. Early correct movement can effectively reduce postoperative adhesion and tendon sliding disorder. However, in the early stage of activity, attention should be paid to the inflammatory edema stage of tendon healing two weeks after reconstruction of a finger tendon rupture. During active movement, the range of motion of the metacarpophalangeal joint should be controlled to ensure an effective 5 mm sliding range of the tendon, and the frequency of finger movement should be considered to avoid high-intensity and high-frequency pulling [11]. Therefore, when making the early finger extensor digitorum dynamic orthotic, it is necessary to limit the range of finger motion and release the limit gradually. The patient should be given adequate guidance in finger rehabilitation while wearing the dynamic orthotic and strictly following the principles of active and passive movement. Four weeks after surgery, the flexion angle and the tension and torque of the wire spring should be adjusted according to the patient's finger healing and joint movement. This can effectively avoid adhesion between the tendon and surrounding tissue, promote tendon healing, and avoid the occurrence of tendon injury. This study found that early preparation and wearing a dynamic orthotic after finger extensor digitorum injury combined with guidance in early finger rehabilitation exercises can effectively improve patients' hand function and prognosis.

## 7. Conclusion

The experimental group used dynamic orthosis after the first postoperative dressing change, strictly implemented the prescribed rehabilitation guidance, completed the corresponding finger rehabilitation training every day, and adjusted the wire tension and torque on a regular basis. The finger joint mobility and functional evaluation of patients in this group after two months were significantly better than those in the static plaster fixation group. This method can effectively relieve patients' post-injury tension, improve their recovery confidence, reduce rehabilitation pain at a later stage, improve rehabilitation value, and avoid postoperative tendon adhesion. This method is worthy of clinical application.

## Figures and Tables

**Table 1:** Comparison of general data between the two groups.

Groups	Gender		Age
	Male	Female	
Group A (*n* = 24)	18	6	43.04 ± 12.55
Group B (*n* = 24)	18	6	42.58 ± 12.86
*P*value	*P* > 0.05		*P* > 0.05

**Table 2:** Standard method.

Observation index	Excellent	Good	General	Poor
TAM	Normal flexion and extension activities	>75% of the healthy finger	50%–75% of the healthy finger	50%–75% of the healthy finger
TPM	Normal flexion and extension activities	>75% of the healthy finger	50%–75% of the healthy finger	50%–75% of the healthy finger

**Table 3:** Changes in TAM and TPM of the injured finger in the two groups ($\bar{x}\pm S$).

Groups	TAM			
	Before treatment	1 month	2 month	*P* value
Group A (*n* = 24)	96.33 ± 10.14	59.38 ± 11.61^a^	126.25 ± 28.45^ab^	*P* < 0.05
Group B (*n* = 24)	96.67 ± 9.23	139.29 ± 16.81^a^	168.79 ± 19.92^ab^	
*P* value	*P* > 0.05	*P* < 0.05	*P* < 0.05	

Groups	TPM			
	Before treatment	1 month	2 month	*P* value

Group A (*n* = 24)	113.42 ± 11.07	107.42 ± 28.13^a^	149.88 ± 29.70^ab^	*P* < 0.05
Group B (*n* = 24)	113.58 ± 10.81	157.08 ± 17.11^a^	188.29 ± 25.62^ab^	
*P* value	*P* > 0.05	*P* < 0.05	*P* < 0.05	

Note: Intragroup comparison of the changes of active and passive movement of injured finger joints between the two groups. *P* < 0.05, the difference was statistically significant compared with before treatment; ^b^*P* < 0.05, the difference was statistically significant compared with the first month of treatment.

**Table 4:** Comparison of superior rate of the injured finger between the two groups after 2 months of treatment.

Groups	TAM				TPM			
	Excellent	Good	General	Poor	Excellent	Good	General	Poor
Group A (*n* = 24)	0	1	11	12	0	3	17	4
Group B (*n* = 24)	4	8	16	0	2	12	10	2
*P* value	*P* < 0.05				*P* < 0.05			

## Data Availability

The datasets used and/or analysed during the current study are available from the corresponding author upon reasonable request.
